# Enzyme Assay Guided Isolation of an α-Amylase Inhibitor Flavonoid from *Vaccinium arctostaphylos* Leaves

**Published:** 2011

**Authors:** Bahman Nickavar, Gholamreza Amin

**Affiliations:** a Department of Pharmacognosy, School of Pharmacy, Shahid Beheshti University of Medical Sciences, Tehran, Iran.; b Department of Pharmacognosy, Faculty of Pharmacy, Tehran University of Medical Sciences, Tehran, Iran.

**Keywords:** α-Amylase inhibition, mellitus, Quercetin, *Vaccinium arctostaphylos*

## Abstract

The management of postprandial hyperglycemia is an important strategy in the control of diabetes mellitus and complications associated with the disease, especially in the diabetes type 2. Therefore, inhibitors of carbohydrate hydrolyzing enzymes can be useful in the treatment of diabetes and medicinal plants can offer an attractive strategy for the purpose. *Vaccinium arctostaphylos* leaves are considered useful for the treatment of diabetes mellitus in some countries. In our research for antidiabetic compounds from natural sources, we found that the methanol extract of the leaves of *V. arctostaphylos* displayed a potent inhibitory activity on pancreatic α-amylase activity (IC_50_ = 0.53 (0.53 – 0.54) mg/mL). The bioassay-guided fractionation of the extract resulted in the isolation of quercetin as an active α-amylase inhibitor. Quercetin showed a dose-dependent inhibitory effect with IC50 value 0.17 (0.16 – 0.17) mM.

## Introduction

Diabetes mellitus is a chronic metabolic disease characterized by elevated blood glucose levels (1, 2). The control of hyperglycemia is critical in the management of diabetes because acute and chronic complications can occur if the blood glucose concentration is not kept in normal levels (3, 4). One therapeutic approach for diabetic patients, especially type 2 diabetes, is to retard the absorption glucose by inhibition of carbohydrate hydrolyzing enzymes, such as *α*-amylase and *α*-glucodiases. The inhibition of the enzymes leads to decrease in the digestion and absorption of carbohydrates, thereby decreasing the postprandial hyperglycemia (1, 5). 

The use of natural products as complementary approaches to existing medications for the treatment of diabetes mellitus is growing in the world and many plant species in different countries are known to have antidiabetic effects (6, 7). Various members of *Vaccinium *genus are one of the most frequently used natural antidiabetic remedies from plant origin (6, 8, 9). It comprises nearly 200 species, most of them are found in the northern hemisphere (10). The genus is represented in Iran only by the species *V. arctostaphylos *L (11). The plant is a resource of traditional Iranian herbal medicine, which is highly recommended for the treatment of diabetes (12). The decoction and/or infusion of the berries and leaves of *V. arctostaphylos *are effectively used for the treatment of diabetes in Iran, Republic of Georgia and Russia (12, 13). 

To the best of our knowledge, there are few studies on antidiabetic properties of *V. arctostaphylos*. Feshani *et al.*, have shown that the extract of *V. arctostaphylos *berries was an effective antihyperglycemic, antioxidant and antihyperlipidemic agent in alloxan-diabetic rats (9). Also, based on the study of Nickavar and Amin, the berries of *V. arctostaphylos *had a potent inhibitory activity on porcine pancreatic *α*-amylase enzyme (14). However, no study has so far been conducted concerning the antidiabetic activity of the plant leaves. This investigation is a continuation of our previous work to study the antidiabetic and *α*-amylase inhibitory activities of *Vaccinium arctostaphylos *and to identify the active constituents of the plant.

## Experimental

*Plant material*

The leaves of *V. arctostaphylos *were collected from the forest region of Asalem in the north of Iran in August 2002.Voucher specimens were deposited in the herabarium of the Faculty of Pharmacy, Tehran University of Medical Sciences, Tehran, Iran (no. 6520 THE). 

*Chemicals*

All of the chemicals used in this study were purchased from Sigma-Aldrich Chemical Co. (France) and/or Merck Company (Germany). 

*Extraction, chromatography and spectroscopy*

The dried and ground leaves (500 g) were extracted with 90% methanol three times by maceration method and then, the extract was concentrated *in vacuo*. The crude extract (27 g) was diluted with water and partitioned with *n*-C_6_H_12_, CHCl_3_ and EtOAc, successively. The inhibitory effects of the crude extract and all fractions were studied on *α*-amylase activity. The ethyl acetate fraction displayed the highest inhibitory activity. The ethyl acetate fraction was subjected to more fractionation by column chromatography on silica gel (70–230 mesh) using petroleum-ether / ethyl acetate system as the eluent and the eluent polarity was increased by increasing the ratio of EtOAc during the process. Fraction F5 which showed a high inhibitory activity, was purified by repeated preparative layer chromatography on coated plates with silica gel (230–400 mesh) using EtOAc/CH_3_COOH/HCOOH/H_2_O (100:11:11:26, v/v/v/v) as the best developing solvent system. Finally, fraction F_5_ yielded the pure active compound 1 (64 mg). The UV light (366 nm) was used for the visualization of bands before and after spraying with AlCl_3_. 

The pure compound was identified on the basis of spectral and chromatographical studies. The UV-Vis spectrum was recorded on a Shimadzu UV-160A spectrophotometer in methanol. The ^1^H-NMR and ^13^C-NMR spectra were taken on a Bruker FT 500 in DMSO-d_6_ and chemical shifts were recorded as *δ*-values. The EI-MS was obtained on an Agilent Technologies 5973 spectrometer. 

*α-Amylase inhibition test*

The *α*-amylase inhibitory activity was determined using the method described previously by Nickavar and Amin (15, 16). Briefly, 1 mL of the porcine pancreatic *α*-amylase enzyme solution (0.5 IU/mL) in 20 mM phosphate buffer (pH 6.9) was incubated with 1 mL of each test (at various concentrations) for 30 min. The reaction was initiated by adding 1 mL of 0.5% soluble potato starch solution and the mixture was incubated for 3 min at 25°C. Then, 1 mL of the color reagent (96 mM 3, 5-dinitrosalicylic acid and 5.31 M sodium potassium tartrate in 2 M sodium hydroxide) was added and the mixture was placed in a water bath at 85°C. After 15 min, the reaction mixture was diluted with distilled water and the absorbance value was determined at 540 nm. Individual blanks were prepared for correcting the background absorbance. In this case, the color reagent solution was added prior to the addition of starch solution and the mixture then placed in the water bath immediately. Controls were representative of the 100% enzyme activity. They were conducted in an identical fashion replacing tests with 1 mL of the solvent. Acarbose, a well-known *α*-amylase inhibitor, was used as positive control. The inhibition percentage of *α*-amylase was assessed by the following formulae:

$I_{á-\mathrm{amylase}}\left(\%\right)=100.(\frac{A_{\mathrm{control}}-A_{\mathrm{sample}}}{A_{\mathrm{control}}})$

where *A*_control _is the absorbance of each control and *A*_sample_ is the net absorbance of each sample. The net absorbance of each sample was calculated by following the equation:

A_sample_=A_test_- A_blank_

where *A*_test_ is the absorbance of each test and *A*_blank_ is the absorbance of each blank .

The *I*_α-amylase_(%) for each sample was plotted against the logarithm of the sample concentration, and a logarithmic regression curve was established in order to calculate the IC_50_ valve. 

## Results and Discussion

The methanol extract of *V. arctostaphylos *leaves showed a dose-dependent inhibitory effect on the *α*-amylase activity [IC_50_ = 0.53 (0.53–0.54) mg/mL] (Table 1). 

**Table 1:** *α*-Amylase inhibitory activities and IC_50_ values of the leaf extract of *V. arctostaphylos *and its active compound quercetin

Concentration	Inhibition (%)^a^	IC_50_^b^
*Leaf extract (mg/mL)*		
1.00	85.13 ± 0.84	
0.80	75.28 ± 1.07	
0.64	60.62 ± 0.90	0.53 (0.53 – 0.54) mg/mL
0.51	36.87 ± 0.91	
0.41	19.12 ± 0.92	
*Quercetin (mM)*		
0.464	72.23 ± 0.86	
0.297	55.30 ± 0.73	
0.190	43.58 ± 1.02	0.17 (0.16 – 0.17) mM
0.122	22.02 ± 0.82	
0.078	10.06 ± 0.90	

^a^ The data are expressed as means ± SEM for five experiments in each group.^ b^ The IC_50 _values were established by logarithmic regression curves with normalized data (using the computer software GraphPad Prism 3.02 for Windows) and presented as their respective 95% confidence limits.

In order to identify the active components, solvent-solvent partition performed with *n*-C_6_H_12_, CHCl_3_ and EtOAc, successively. The ethyl acetate fraction revealed the highest activity therefore; it was selected for further separation. The chromatographical analysis of the ethyl acetate fraction showed flavonoid compounds. The most active flavonoid compound was isolated as the pale yellow amorphous powder (64 mg). It had R_f _= 0.48 on TLC (silica gel 60) with EtOAc/CH_3_COOH/HCOOH/H_2_O (100:11:11:26, v/v/v/v). The spectroscopic data for the compound were as follows: 

UV-Vis: λ_max_ (in CH_3_OH) = 260, 275 (shoulder), 380 nm; + AlCl_3_ = 265, 455 nm; + AlCl_3_ + HCl = 265, 425; + NaOAc = 275, 380 nm (degradation); + NaOAC + H_3_BO_3_: 260, 395 nm; + NaOMe = rapid degradation.

^1^H-NMR (500 MHz, in DMSO-d_6_), δ: 6.15 (1H, d, *J *= 1.8 Hz, H-6), 6.39 (1H, d, *J *= 1.8 Hz, H-8), 6.88 (1H, d, *J *= 8.4 Hz, H-5’), 7.53 (1H, dd, *J *= 8.4, 1.9 Hz, H-6’), 7.65 (1H, d, *J *= 1.9 Hz, H-2’).

^13^C-NMR (125 MHz, in DMSO-d_6_), δ: 94.2 (C-8), 99.0 (C-6), 103.8 (C-10), 115.9 (C-2’), 116.5 (C-5’), 120.8 (C-6’), 122.8 (C-1’), 136.5 (C-3), 145.9 (C-3’), 147.6 (C-2’), 148.5 (C-4’), 157.0 (C-5), 161.5 (C-9), 164.8 (C-7), 176.0 (C-4). 

EI-MS (70 eV), *m*/*z *(*I *%): 302 (100%) (M^+^). 

**Figure 1:**
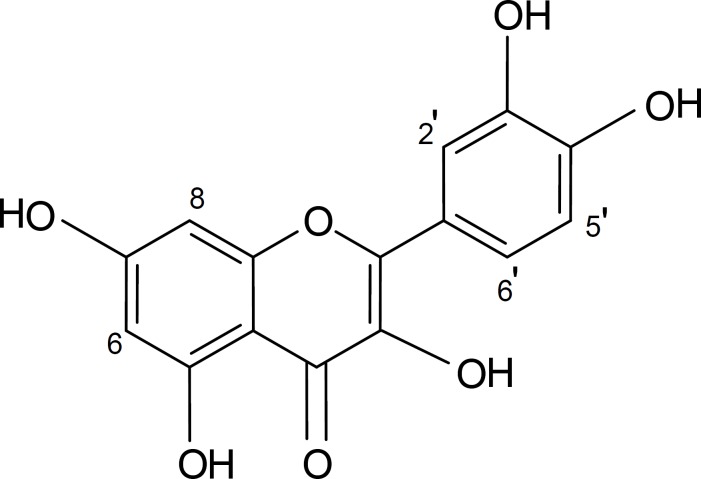
Chemical structure of quercetin

The spectral data of the compound showed that it was quercetin (Figure 1) and all of its data were matched with those reported in the literature (17) .

In this study, quercetin inhibited *α*-amylase activity in a dose-dependent manner. The IC_50 _values for *α*-amylase inhibition by quercetin and acarbose (as the positive control) were 0.17 (0.16 – 0.17) mM and 0.033 (0.031-0.036) mM, respectively (Figure 1 and Table 1). 

The genus of *Vaccinium *generally produces a variety of phenolic metabolites especially anthocyanins, flavonols, phenolic acids, procyanidines, *etc *(18). Quercetin has already been isolated from leaves of some species belonging to the genus *Vaccinium *such as *V. reticulatum *and *V. calycinum*, *V. myrtillus*, *V. angustifolium*, *V. vitis-idaea*, *etc *(19-22). Phytochemical studies on the different parts of *V. arctostaphylos *show the occurrence of flavonoids and coumarins in leaves, phenolic acids and their derivatives in leaves and unripe berries and anthocyanins in ripe berries (23-30) . However, the study is the first report on the inhibitory effect of *V. arctostaphylos *leaves on *α*-amylase and isolation of quercetin as their active component. 

Based on these experimental results, it can be concluded that a part of antidiabetic effects observed from *V. arctostaphylos *leaves might be due to the inhibition of the *α*-amylase by the flavonoid quercetin.
